# Regulation of cerebral arterial BKCa channels by angiotensin II signaling in adult offspring exposed to prenatal high sucrose diets

**DOI:** 10.1042/BSR20160624

**Published:** 2017-06-21

**Authors:** Xiuxia Gu, Axin He, Xiaorong Fan, Ruixiu Shi, Xueqin Feng, Le Bo, Lin Jiang, Na Li, Jue Wu, Yuxian Yang, Qinqin Gao, Zhice Xu

**Affiliations:** 1 Institute for Fetology, the First Affiliated Hospital of Soochow University, Suzhou, China; 2 Center for Perinatal Biology, Loma Linda University, California, U.S.A.; 3 Department of Obstetrics and Gynecology, Suzhou Science & Technology Town Hospital, Suzhou Hospital Affiliated to Nanjing Medical University, China

**Keywords:** Adult offspring, Angiotensin II, BKCa channels, Middle cerebral artery, Prenatal high sucrose diets

## Abstract

Prenatal insults have been shown to affect vascular functions, leading to increased risks of cardiovascular diseases in offspring. The present study determined whether high sucrose (HS) intake in pregnancy affected central vascular functions in middle cerebral artery (MCA) of offspring. Sprague-Dawley rats were fed a standard food and tap water with normal or high (20%) sucrose content during pregnancy. Offspring were maintained with normal diets and tap water. Central vascular functions and related ion channels were assessed in male offspring at 5 months old. Compared with the control, angiotensin II (AII)-induced vasoconstrictions were significantly higher in the MCA of the offspring exposed to prenatal HS. In the MCA, large conductance Ca^2+^-activated K^+^ channels (BKCa) currents were decreased with a reduction of opening frequency, sensitivity to intracellular Ca^2+^/membrane voltage, and BKβ1 expression. mRNA levels of AT1α and AT2, as well as AT1/AT2 ratio, were significantly increased in the MCA of offspring following exposure to prenatal HS diets. The data suggested that prenatal HS diets could alter microvascular activities in the MCA, probably via changes of BKCa channels in the brain.

## Introduction

The hypothesis of ‘fetal programming of adult diseases’ proposed by Barker postulated that impaired *in utero* growths are determinants of diseases in adult life [1]. From animal studies, a number of prenatal insults (including exposure to caffeine, hypoxia, and high salt diet) could affect vascular reactivity, which may result in adverse cardiovascular outcomes in the offspring [2–4]. It is well known that vascular smooth muscle cells (VSMCs) play a key role in the control of vascular tone. Changes in VSMCs membrane potential regulated by K^+^ channels have been proposed to be involved in modulating physiological and pathophysiological vessel tone [5–7]. To date, four distinct types of K^+^ channels have been identified in VSMCs: voltage-dependent K^+^ channels, Ca^2+^-activated K^+^ (K_Ca_) channels (including the large (BKCa), intermediate (IKCa), and small (SKCa) conductance subfamily), inward rectifier K^+^ channels, and ATP-sensitive K^+^ channels [7]. Among above K^+^ channels, BKCa channels are key subgroups to regulate membrane electrical events in virtually all VSMCs. Activation of BKCa channels can lead to hyperpolarization of cell membrane and provide a negative feedback in the modulation of vascular tone [8,9].

A long-term health consequence in offspring may be induced by maternal unbalanced diets during pregnancy. Feeding mother rats with a diet high in sugar could cause adverse outcomes such as cardiovascular diseases and metabolic syndrome in the offspring [10,11]. Our previous study demonstrated that high sucrose (HS) diets during pregnancy not only altered microvessel tone via depressed BKCa channels in offspring’s mesenteric arteries [12], but also led to impaired learning and memory in the male offspring [13]. Since middle cerebral artery (MCA) is a critical blood vessel in supplying blood and oxygen to the brain, MCA dysfunction could be considered as major contributor to multiple brain problems, such as vascular dementia and cognitive dysfunction [14,15]. Consequently, the reported evidence led us to hypothesis that HS diets during pregnancy might increase risks of central vascular dysfunction resulting from altered BKCa channels functions in the MCA.

## Materials and methods

### Experimental animals

Pregnant Sprague-Dawley rats (10–12 weeks old) were from Animal Center of Soochow University. All rats were housed in a controlled environment, 22–23°C and a 12-h light/dark cycle. Rats were randomly divided into the control and HS group. Control group was provided with fresh tap water and standard rat food, while HS group was given the same food with 20% sucrose solution from gestational day 1 to 21. At gestational day 21, some of pregnant rats were killed for fetal studies, others were allowed to give birth naturally. After delivery, newborn rats were kept with their mothers until weaning. The male offspring were fed with standard rat food and tap water in the same environment until 5 months old. All experimental procedures were approved by the Institutional Animal Care Committee and conformed to the Guide for the Care and Use of Laboratory Animals (NIH Publication No. 85-23).

### Blood glucose, body, and brain weight

After an 8-h fasting period, pregnant rats (on gestational day 21) as well as the male offspring were anesthetized with sodium pentobarbital (100 mg/kg; Hengrui Medicine, Jiangsu, China). Fetal, offspring’s body and brain weight were determined. The ratio of brain to body weight was calculated. Maternal and offspring’s blood samples were collected from the abdominal aorta. Fetal blood samples were also obtained following decapitation. Blood samples were collected into ice-cold plastic tubes with anticoagulant and centrifuged at 3000 rpm for 10 min. Plasma was transferred to new tubes, and stored at −80°C for measuring blood glucose. Fasting blood glucose levels were determined by a Nova analyzer (Nova Biomedical, Waltham, MA, U.S.A.).

### Measurement of vessel tone

Immediately after killing of male offspring rats, MCA was rapidly excised and placed in the 4°C cold physiological salt solution containing (mmol/l; NaCl, 130; KCl, 4.7; NaHCO_3_, 14.9; KH_2_PO_4_, 1.18; MgSO_4_·7H_2_O, 1.18; CaCl_2_·2H_2_O, 1.56; EDTA, 0.026; glucose, 5.5; and HEPES 10.0; pH 7.4), gassed continuously with 5% CO_2_ in O_2_. Rings of MCA were carefully prepared, and mounted in a multimyograph system (Danish Myo Technology, Midtjylland, Denmark) for recording of isometric tension [3,12]. The vessel rings were given an initial tension and were equilibrated for 60 min. KCl (60 mmol/l) was used to achieve optimal resting tension before adding drugs. Contractile capacity of the drugs was normalized by comparing with the contraction elicited by KCl alone. The vessel rings were contracted with cumulatively concentrations of angiotensin II (AII, 10^−11^–10^−6^ mol/l) in the presence or absence of losartan (AT1R antagonist, 10^−5^ mol/l), or PD123319 (AT2R antagonist, 10^−5^ mol/l) as reported [16]. All drugs were purchased from Sigma (St. Louis, U.S.A.).

### Electrophysiological measurements

#### Whole-cell recording

Myocytes were isolated enzymatically from dissected MCA [2,12]. Briefly, MCA were first dissected gently and cut into small fragments (approximately 0.5 mm) in oxygenated ice-cold physiological solution (PSS) (mmol/l: NaCl, 120.9; NaHCO_3_, 25.0; KCl, 4.6; NaH_2_PO_4_, 1.2; MgCl_2_, 1.2; CaCl_2_·2H_2_O, 2.8; and glucose, 5.0; pH 7.4), and then were placed for 35 min at 37°C in PSS solution containing 5 mg/ml papain, 2 mg/ml ABV, and 1 mg/ml dithiothreitol. Single cells were obtained by gentle trituration with a wide-bore glass pipette, stored at 4°C and used within 6 h. Whole-cell K^+^ currents were recorded with whole-cell patch-clamp technique using an Axon Multiclamp 700B with Clampex 10.2 (Axon Instruments, Foster City, CA, U.S.A.) as previously described [2]. Electrode resistances were 3–5 MΩ when filled with pipette internal solution (mmol/l: K-Asp, 110; KCl, 30; EGTA, 1; Na_2_ATP, 3; CaCl_2_, 0.85; glucose, 10; and HEPES, 10; pH 7.2 with KOH). The bath solution contained (mmol/l: NaCl, 135; KCl, 5; MgCl_2_, 1; CaCl_2_, 1.8; glucose,10 and HEPES,10; pH 7.4 with NaOH). Whole-cell K^+^ currents were achieved by a series of 500 ms depolarizing voltage steps. Voltage steps were made at 10 mV increments to +60 mV from a holding potential of −60 mV. Whole-cell K^+^ currents were monitored before and after adding iberiotoxin (IBTX, a selective BKCa inhibitor, 0.1 μmol/l) or/and AII (10^−5^ mol/l) for 30 min. The IBTX sensitive currents (BKCa) were obtained by subtracting residual current after adding IBTX from the whole-cell K^+^ currents. All the results in Figure 3 were obtained under whole-cell configuration.

#### Single channel recording

Myocytes were placed in a chamber with a volume of 1 ml. Single channel currents were recorded from inside/out (I/O) patches. The pipette solution contained (mmol/l: KCl, 145; EGTA, 1; HEPES, 10; and glucose, 5; pH 7.3–7.4 with KOH). The bath solution contained (mmol/l: KCl, 145; EGTA, 1; HEPES, 10; and glucose, 5; pH 7.3–7.4 with KOH). BKCa single channel currents were monitored as previously described [17]. Average activity of BKCa channels was measured as mean open probability (Po). Voltage dependent behavior of the single channel Po was modeled with the Boltzmann function [17]. In the calcium-dependent test, various [Ca^2+^]i (intracellular Ca^2+^, 0.1, 1, and 3 μM) within the range of physiological concentrations were used [2]. Free Ca^2+^ in solution was adjusted to the desired level by adding CaCl_2_ (determined using WinMAXC software; Chris Patton, Stanford University). EGTA (10 mM) was added into the bath solution to ensure an absolute Ca^2+^ free condition [18,19]. The Ca^2+^-dependent activation was fitted with the Hill equation [17]. The effect of tamoxifen on single BKCa channel currents was also studied. BKCa currents on single myocyte were recorded in inside/out patches before and after adding tamoxifen (10^−5^ mol/l) at +40 mV with 10^−6^ mol/l [Ca^2+^]i [2]. BKCa currents in Figure 4 were obtained using single-channel recording. All electrophysiological studies were carried out at room temperature. All drugs were purchased from Sigma–Aldrich.

### Quantitative real-time PCR (qRT-PCR)

Total RNA was extracted from MCA of male offspring using TRIzol reagents (Invitrogen Co., Ltd. Carlsbad, CA, U.S.A.), and first-strand cDNA was synthesized using reverse transcription kits (Takara Biotechnology Co., Ltd. Dalian, China). mRNA abundance of AT1α, AT1β, AT2R, BKα, and BKβ1 was determined by real-time PCR using an iCycler thermal cycler and iQ software (Bio-Rad Laboratories, Hercules, California). ΔΔ*C*_t_ method was used to comparatively quantify the amount of mRNA level [20]. All the primers used were listed in Table 1.

**Table 1:** The list of the primers used in the present study

qRT-PCR primers	Sequence	
Gene name	5′-3′	3′-5′
AT1α	CTTCTCAATCTCGCCTTGGC	AAGGAACACATGGCGTAGA
AT1β	GGTTCAAAGCCTGCAAGTGA	CGGTTAACAGTGGCTTTGCT
AT2	TGGCTTCCCTTCCATGTTCT	TCTCTCTCTTGCCTTGGAGC
BKα	GCTGACTGCAGCTGGATTCAT	CTGTAGACATTGTGACCATGAG
BKβ1	CTAAATGACTGTTGCCTCCTG	AGGATGTAGTAAGTGATGGCGG

### Data analysis

Data were expressed as mean ± S.E.M. Statistical significance (*P*<0.05) was determined by *t*-test or two-way ANOVA analysis followed by Bonferroni. Curve fitting was performed with SigmaPlot 10 (Systat Software, Inc., Chicago, IL, U.S.A.) and GraphPad Prism 5 (GraphPad Software, Inc., San Diego, CA, U.S.A.).

## Results

### Body, brain weight, and plasma glucose

**Figure 1:**
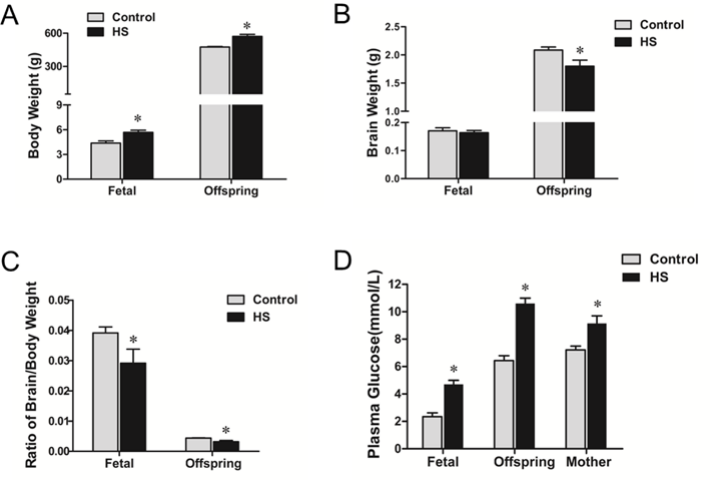
Body, brain weight, and plasma glucose. (**A**–**C**) Body weight, brain weight, and the ratio of brain/body weight in the fetuses and offspring. (**D**) Plasma glucose in the fetus, offspring, and mothers; *P <0.05; *N*=10/each group.

Both fetal and adult offspring’s body weight in HS group were increased compared with the control group (*P*<0.05; Figure 1A). There were no significant differences in brain weight between the control and HS group at fetal period (*P*>0.05; Figure 1B), while brain weight of adult offspring (5 months old) was significantly decreased in the HS group (*P*<0.001; Figure 1B). The ratio of brain to body weight in the HS offspring at both ages was significantly lower than that of the control (*P*<0.05; Figure 1C). In addition, fetal and offspring’s, as well as maternal plasma glucose in the HS group was significantly increased compared with that in the control (*P*<0.01; Figure 1D).

### Angiotensin II-induced contractions in the middle cerebral artery

**Figure 2:**
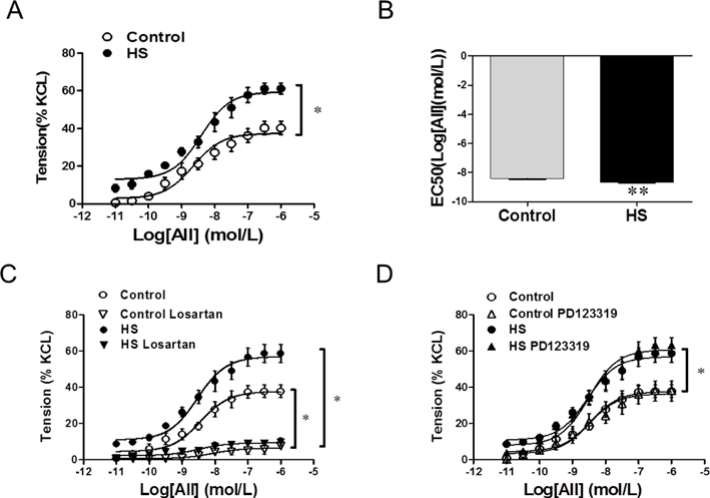
Angiotensin II-induced contractions in the middle cerebral artery. (**A** and **B**) Response curves and EC_50_ values of AII-induced contractions. (**C** and **D**) Response curves of AII-induced contractions in presence of losartan or PD123319. Losartan, an AT1R antagonist; PD123319, an AT2R antagonist; AII, angiotensin II; *P <0.05, **P<0.01; *N*=8, *n*=25 for each group; *N*, number of offspring; *n*, number of vascular rings.

Compared with that in the control, the maximal response to AII was significantly increased in MCA from the HS offspring. AII-induced maximum contraction was 39.20 ± 4.448% in control and 63.40 ± 6.718% in HS group (*P*<0.05, Figure 2A). The EC_50_ (log [AII](mol/l)) was −8.408 ± 0.1114 in control and −8.648 ± 0.1434 in HS group (*P*<0.05, Figure 2B). AT1R (type 1 receptor of AII) inhibitor, losartan, completely inhibited AII-induced vasoconstrictions in both the control and HS group, while AT2R (type 2 receptor of AII) inhibitor, PD123319, showed no effect (Figure 2C and D).

### The effect of angiotensin II on BKCa channels

**Figure 3:**
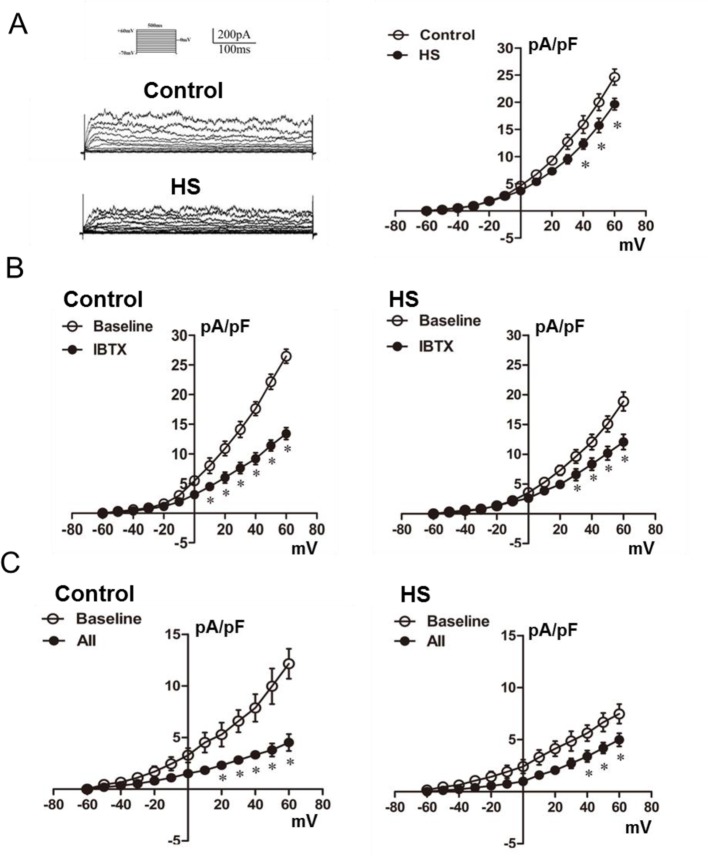
Whole-cell K^+^ currents and the effect of angiotensin II on BKCa channels. (**A**) K^+^ currents at baseline and current–voltage (*I*–*V*) curves of K^+^ currents in the control and HS offspring (*N*=8, *n*=18 for control; *N*=8, *n*=20 for HS). (**B**) K^+^ currents at baseline and after adding IBTX (0.1 μmol/l, *N*=9, *n*=20 for each group). (**C**) BKCa currents at baseline and after adding AII (10^−5^ mol/l, *N*=9, *n*=23 for each group). IBTX, a selective BKCa inhibitor; AII, angiotensin II; *P<0.05; *N*, number of offspring; *n*, number of myocytes.

As shown in Figure 3A, whole-cell K^+^ currents were decreased at +40 to +60 mV in the HS myocytes compared with the control. A selective BKCa inhibitor, IBTX, reduced the whole-cell K^+^ currents in the HS myocytes (from 18.86 ± 1.58 pA/pF at baseline to 12.07 ± 1.29 pA/pF, TP = +60 mV, *P*<0.05) and the control cells (from 26.45 ± 1.19 pA/pF at baseline to 13.40 ±1.01 pA/pF, TP = +60 mV, *P*<0.05, Figure 3B), indicating that BKCa currents that were subtracted from the baseline by IBTX were decreased in HS myocytes compared with that in control. BKCa currents were also decreased in HS myocytes (7.46 ± 0.93 pA/pF at baseline, TP = +60 mV, Figure 3C) compared with the control (12.15 ± 1.44 pA/pF at baseline, TP = +60 mV, Figure 3C). In addition, AII caused significant inhibition of BKCa currents in the control (from 12.15 ± 1.44 pA/pF at baseline to 4.52 ± 0.36 pA/pF, TP = +60 mV, *P*<0.05, Figure 3C). In MCA myocytes from the HS offspring, BKCa currents were 7.46 ± 0.93 pA/pF at baseline and 4.98 ± 0.62 pA/pF after pretreating with AII (10^−5^ mol/l, TP = +60 mV, *P*<0.05, Figure 3C). The decrease in BKCa currents by AII in the HS offspring was less than that in the control, indicating a decreased suppression of BKCa currents by AII in the HS offspring.

### BKCa channels in the middle cerebral artery

**Figure 4:**
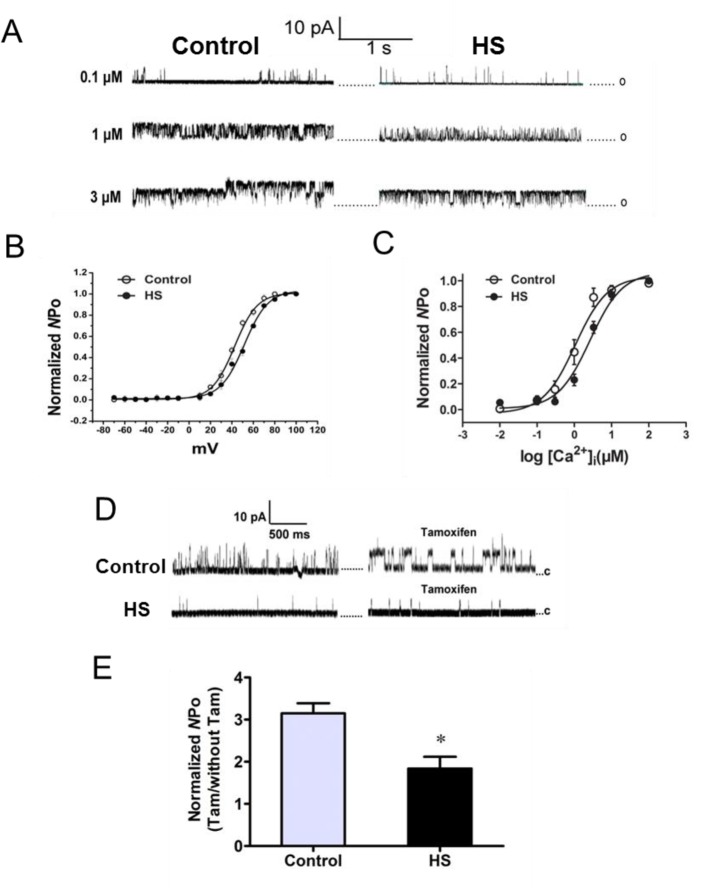
BKCa channels in the middle cerebral artery. (**A**) Representative recordings of single BKCa channels at a membrane voltage of +40 mV from the control and HS myocytes exposed to increasing [Ca^2+^]i (0.1, 1, and 3 μM). (**B**) Voltage dependence of BKCa channels in [Ca^2+^]i at 10^−6^ mol/l (*N*=6, *n*=20 for each group). (**C**) Ca^2+^ sensitivity of BKCa channels from the control and HS myocytes (*N*=6, *n*=18 for each group). (**D**) Representative recordings of single BKCa channels (HP = +40 mV; [Ca^2+^]i = 10^−7^mol/l) from the control and HS myocytes exposed to tamoxifen (Tam). (**E**) Bar plot summarizes the mean SEM fold change in the NPo of BK channels after the application of Tam (*N*=7, *n*=22 for each group). *P<0.05; *N*, number of offspring; *n*, number of myocytes.

We next examined the differences in the functional properties of BKCa channels, which may underlie the difference in whole BKCa currents amplitude between control and HS myocytes. Ca^2+^/voltage sensitivity of BKCa channels was determined first. With an increase in [Ca^2+^]i concentration, both groups showed an enhanced NPo (normalized to the max probability) of BKCa channels (Figure 4A), while the Po-[Ca^2+^]i curve for the HS group was shifted right with an increased *K*_d_ (open half of the channels) value (control versus HS: 1.07 ± 0.10 versus 2.65 ± 0.07, Figure 4C). We then examined the voltage sensitivity of BKCa channels in the presence of 10^−6^ mol/l [Ca^2+^]i (Figure 4B). There was a significantly right shift for BKCa channels in the HS myocytes with an increased half-activation potential values (*V*_1/2_) (control versus HS: 42.63 ± 0.55 mV versus 57.08 ± 1.79 mV). These data indicated that a decreased Ca^2+^/voltage sensitivity of BKCa channels in HS myocytes compared with that in the control. Then, the effects of tamoxifen on BKCa channels were examined in the control and HS myocytes. Tamoxifen could significantly increase the activity of BKCa channels when it was associated with BKβ1-subunit [21,22]. Application of tamoxifen evoked nearly a 3.15-fold increase in the NPo of BKCa channels in the control group, while only 1.8-fold increase in HS group (*P*<0.05, Figure 4D and E), indicating that the reduced BKCa channels activity in HS myocytes was related to BKβ1-subunit.

### mRNA levels of angiotensin II receptors and BKCa channels subunits

**Figure 5:**
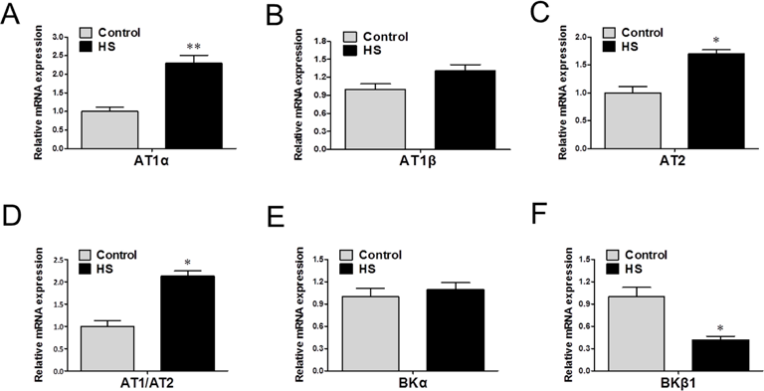
mRNA levels of angiotensin II receptors and BKCa channels subunits. (**A-F**) mRNA levels of AT1α, AT1β, AT2, BKα, and BKβ1 in offspring’s MCA. *P<0.05, *N*=20 for each group; *N*, number of offspring.

mRNA levels of AT1α and AT2 were increased in the MCA of HS group (*P*<0.05, Figure 5A and C), whereas no significant differences in AT1β (Figure 5B), resulting in an increase in the AT1R/AT2R ratio in the HS group (*P*<0.05, Figure 5D). mRNA levels of BKCa channels subunit BKβ1, not BKα, were significantly decreased in the HS group compared with that in the control (Figure 5E and F).

## Discussion

It is known that prenatal insults may have long-term impact on health after birth. The present study demonstrated that prenatal HS diets could affect fetal development *in utero*, and increase risks of cerebral dysfunction in the adult offspring via adverse vascular functions in the MCA. New information obtained include: first, although no significant difference was found in brain weight between the two groups at fetal period, brain weight of HS adult offspring was less than that in the control. Comparing with the control group, higher body weight and lower ratio of brain to body weight, as well as higher plasma glucose levels of HS offspring were noted. These results indicated that prenatal HS could influence glucose metabolism and growth development in the offspring. Second, prenatal HS altered the expression of renin–angiotensin system-related receptors, and increased the sensitivity to AII in the MCA of HS offspring. Third, dysfunction of BKCa channels by reduced sensitivity of β1-subunit responses to Ca^2+^/voltage in HS offspring may contribute to enhancing AII-induced vasoconstrictions via a more depolarized membrane potential.

In the present study, brain weight as well as the ratio of brain to body weight were significantly lower in the HS male offspring, suggesting that there existed long-term effects after birth following exposure to prenatal HS diets, and that the adverse effects may extend to important organs such as the brain. In our previous study, maternal HS diets during pregnancy led to impaired learning and memory in the male offspring [13]. These findings indicated that similar to mater obesity and high fat diet [23–25], maternal HS diets during pregnancy could lead to altered fetal neuroendocrine development, subsequently resulting in potential cognitive dysfunction in the offspring.

AII plays a key role in the regulation of cardiovascular homeostasis mainly through binding to type 1 (including AT1α and AT1β) and type 2 (AT2R) receptors [26]. AT1R is a major subtype mediating vasoconstrictions, whereas AT2R could counteract vasoconstrictions [27,28]. In our microvessel experiments, the curve of AII-induced concentration-dependent constrictions were altered in HS group with increased *E*max and decreased EC_50_, indicating that AII-induced constrictions in HS group were associated not only with number of AII receptors but also with the sensitivity of the receptors to AII. AT1R-specific antagonist could block AII-induced constrictions, whereas the AT2R blocker showed no significant effects, suggesting that the difference of AII-mediated vasoconstrictions between HS and control was due to the AT1R. This was further supported by the molecular assay. Compared with the control, AT1R and AT2R expression, as well as the ratio of AT1R to AT2R, was significantly increased in the HS group. These results indicated that AII-induced vasoconstrictions in HS group were closely related to the up-regulated AT1R.

To determine possible mechanisms underlying the enhanced sensitivity of AT1R to AII, we considered ion channels on smooth muscle cells of the MCA in the offspring. AT1R is a G-protein-coupled receptor that can suppress K^+^ channels by interacting with BKCa channels and inhibits their activity [29]. Previous studies suggested that AII mediated-pressor responses were linked to a direct inhibition of BKCa channels, resulting in membrane depolarization and smooth cell contraction [30,31]. As a major class of K^+^ channels, BKCa include a pore-forming α-subunit and a regulatory β-subunit (including four isoforms (β1–4)) [32]. The α-subunit maintains basic structure of BKCa channels. Of the four isoforms, β1 subunit is the predominant isoform in VSMCs and modifies functional properties of α-subunit, including increasing channel Ca^2+^/voltage sensitivity [32–37]. Activities of BKCa channels can lead to membrane hyperpolarization, which in turn contribute to counteracting smooth cell depolarization and constriction [33,37]. Contrarily, the reduction in BKCa channel activity due to channel dysfunction in vascular smooth cells can blunt the negative regulation of vessel tone [33,37,38]. Recent studies have shown that BKCa channels have a pathophysiological role in the development of adverse cardiovascular outcomes following exposure to prenatal insults [12,16]. Our vasoconstriction and electrophysiological data suggested that AII-induced vasoconstrictions in HS offspring’s MCA were partially attributed to the reduced BKCa channel activity, which could be explained in either of two ways: (1) as shown in whole-cell K^+^ currents data, the baseline of whole-cell K^+^ currents as well as the decreased K^+^ currents by the BKCa channels inhibitor were reduced in HS group, indicating that BKCa currents in HS offspring’s MCA were less than that in the control. Based on the further results from PCR and single-channel K^+^ currents analysis, BKCa currents were decreased because both the opening frequency of BKCa channels and the sensitivity of BKCa channels to Ca^2+^ were reduced that was linked to a decreased expression of BKβ1. Less active BKCa channels in the HS group could not offset the AII-mediated constrictions as intracellular Ca^2+^ increases in response to AII so that the cell membrane was hard to repolarization. (2) In the present study, the suppressed BKCa currents by AII were lower in HS offspring than that in the control, suggesting that AII-induced BKCa mediated greater membrane depolarization in HS offspring so that AII-produced vasoconstrictions were stronger. In addition, the reduced BKCa channels activity at rest in HS group would also likely lead to a more depolarized membrane potential in the latter case, closer to threshold for contraction in response to AII. Following studies should consider using specific blockers such as IBTX for further investigation.

In summary, the present study provided new information on the impact of HS intake during pregnancy on fetal programming of vascular diseases. Prenatal HS caused a higher sensitivity to AII in the offspring’s MCA, which might be due to an increased expression of AT1R and reduced activities of BKCa channels. Together, new information achieved from the present study at tissue and cellular levels strongly suggested that the risks in the development of brain problems could be increased following exposure to HS intake in early developmental stages.

## Abbreviations

AII: angiotensin II
BKCa: Large conductance Ca^2+^-activated K^+^ channels
HS: high sucrose
MCA: middle cerebral artery
VSMC: vascular smooth muscle cell
